# Long non‐coding RNA as potential diagnostic markers for acute myeloid leukemia: A systematic review and meta‐analysis

**DOI:** 10.1002/cam4.7376

**Published:** 2024-06-12

**Authors:** Yenealem Solomon, Ayenew Berhan, Andargachew Almaw, Tamirat Ersino, Shewaneh Damtie, Teklehaimanot Kiros, Alemie Fentie, Ermias Sisay Chanie, Anteneh Mengist Dessie, Ermiyas Alemayehu

**Affiliations:** ^1^ Department of Medical Laboratory Science, College of Health Sciences Debre Tabor University Debre Tabor Ethiopia; ^2^ School of Medical Laboratory Science, College of Health Science Wolaita Sodo University Wolaita Sodo Ethiopia; ^3^ Department of Pediatrics and Child Health Nursing, College of Health sciences Debre Tabor University Debre Tabor Ethiopia; ^4^ Department of Public Health, College of Health Sciences Debre Tabor University Debre Tabor Ethiopia; ^5^ Department of Medical Laboratory Sciences, College of Medicine and Health Sciences Wollo University Dessie Ethiopia

**Keywords:** acute myeloid leukemia, biomarker, diagnosis, long noncoding RNA, meta‐analysis

## Abstract

**Background:**

Acute myeloid leukemia (AML) is aggressive type of hematological malignancy. Its poses challenges in early diagnosis, necessitating the identification of an effective biomarker. This study aims to assess the diagnostic accuracy of long noncoding RNAs (lncRNA) in the diagnosis of AML through a meta‐analysis. The study is registered on the PROSPERO website with the number 493518.

**Method:**

A literature search was conducted in the PubMed, Embase, Hinari, and the Scopus databases to identify relevant studies. We pooled sensitivity, specificity, positive likelihood ratio (PLR), negative likelihood ratio (NLR), diagnostic odds ratio (DOR), and area under the summary receiver operating characteristics (ROC) using Stata 14.1 software. Heterogeneity between studies was determined through the *I* ^2^ statistic and Cochran‐Q test. A random effect model was chosen due to significant heterogeneity among included studies. Meta‐regression and subgroup analysis were performed to assess the potential source of heterogeneity. Furthermore, potential publication bias was estimated using Deek's funnel plot asymmetry test.

**Results:**

A total of 14 articles covering 19 studies were included in this meta‐analysis comprising 1588 AML patients and 529 healthy participants. The overall pooled sensitivity, specificity, PLR, NLR, DOR, and the area under the summary ROC curve were 0.85 (95% CI = 0.78–0.91), 0.82 (95% CI = 0.72–0.89), 4.7 (95% CI = 2.9–7.4), 0.18 (95% CI = 0.12–0.28), 26 (95% CI = 12–53), and 0.90 (95% CI = 0.87–0.93), respectively. Moreover, lncRNAs from non‐bone marrow mononuclear cells (BMMC) had superior diagnostic value with pooled sensitivity, specificity, and AUC were 0.93, 0.82, and 0.95, respectively.

**Conclusion:**

This meta‐analysis demonstrated that circulating lncRNAs can serve as potential diagnostic markers for AML. High accuracy of diagnosis was observed in non‐BMMC lncRNAs, given cutoff value, and the GADPH internal reference gene used. However, further studies with large sample size are required to confirm our results.

## 1 INTRODUCTION

Acute myeloid leukemia (AML) is an aggressive type of hematological malignancy characterized by the uncontrolled proliferation and impaired differentiation of the clonal myeloid progenitors.^1^ It is a complex disease with diverse pathophysiologic, cytologic, clinical, and molecular profiles. The etiology of AML is multifactorial, involving both genetic and environmental factors.^1^, ^2^

AML is the most common type of leukemia in adults, accounting for 80% of acute leukemias and 18% of all leukemias.^3^ AML cases are increasing globally, with high morbidity and mortality in older patients.^3^ The incidence rate of AML is 4.1 cases per 100,000 people per year. In 2023, it is estimated that there were 11,310 deaths attributed to AML.^4^ The incidence of AML is directly correlated with increasing age.^5^, ^6^

Long noncoding RNAs (lncRNA) are transcripts of over 200 base pairs that cannot be translated into functional proteins because they lack open reading frames.^7^, ^8^ Through the advent of high‐throughput technology, it has been discovered that the majority of the human genome is made up of noncoding genes. In contrast, only 2% of the genome consists of coding genes.^9^ lncRNA regulates a range of vital cellular functions.^10^ They exert their function through interaction with RNA, DNA, and proteins. Their main role is the control of gene expression, which can be achieved via post‐transcriptional mechanisms, chromatin alteration, or transcription factor regulation.^11^

Various studies indicated that lncRNAs play both structural and functional roles in AML, including proliferation, differentiation, activation, and initiation of apoptosis in different cell types.^12^, ^13^, ^14^ By regulating gene expression at pre‐transcriptional, post‐transcriptional, and epigenetic stages, lncRNAs significantly influence AML development and drug resistance.^13^ Dysregulation of lncRNAs in AML promotes differentiation block, proliferation, and therapeutic resistance, and they can serve as tumor suppressors or biomarkers.^15^ Additionally, lncRNAs promote AML cell proliferation through various mechanisms like modulating leukemic cell metabolism, translational regulation, and protein synthesis, while also promoting differentiation block in AML cells.^15^

Specific patterns of lncRNA expression associated with different AML subtypes suggest that lncRNAs are crucial in the disease's pathogenesis and treatment.^16^ The dysregulation of lncRNAs in AML plays a significant role in disease pathogenesis, progression, and treatment.^12^, ^15^ The significance of lncRNA dysregulation in AML is substantial, offering valuable insights for clinical diagnosis, prognosis, and targeted therapy for AML patients.^14^, ^17^, ^18^ Due to their differential expression, lncRNAs can serve as predictors of disease stages.^18^, ^19^ Moreover, the dysregulation of lncRNAs in AML provides valuable insights for clinical diagnosis, prognosis, and targeted therapy.^20^

Currently, AML diagnosis is based on the presence of blast cells in either bone marrow (BM) or blood, along with morphology, immunophenotype, molecular genetics, and cytogenetics.^21^ These diagnostic techniques are invasive; obtaining BM samples involves a lengthy and painful procedure for the patient. In addition, early diagnosis of AML is difficult due to the delayed onset of symptoms and the late spread of leukemic blast cells to the peripheral blood.^22^ Interestingly, lncRNAs have been found in various body fluids, including serum, plasma, saliva, urine, and tissues, making them suitable markers for AML diagnosis. Moreover, lncRNA detection is both noninvasive and highly specific, positioning lncRNAs promising as diagnostic indicators, predictive biomarkers, and potential therapeutic targets in cancer.^23^, ^24^, ^25^

Despite conflicting findings in previous studies, the exact reason for this inconsistency remains unclear. Furthermore, reliable conclusions regarding diagnostic accuracy of lncRNAs in AML patients have not been drawn. To address this gap in the literature, we conducted the first ever diagnostic meta‐analysis to ascertain the diagnostic accuracy of circulating lncRNAs in AML patients.

## 2 MATERIALS AND METHODS

This systematic review and meta‐analysis was conducted following the guidelines outlined in the Preferred Reporting Items for Systematic Reviews and Meta‐analysis (PRISMA) 2020 guideline (File S1).^26^ The study's protocol was preregistered in PROSPERO with ID 493518.

### 2.1 Literature searching strategy

A comprehensive search was conducted in PubMed, Embase, Hinari, and the Scopus database from July 15, 2023, to August 15, 2023, to identify relevant studies. The reference lists of pertinent articles were checked. Additionally, Gray literature searches were performed using Google and Google Scholar, and the bibliographies of identified studies reviewed to include any additional relevant studies that might have been missed during the electronic database search. Our search encompassed studies written in the English language with no restriction in publication year.

An inclusive literature search was conducted to retrieve reports on the diagnostic value of circulating lncRNAs for patients with AML. The databases were systematically searched in accordance with the Medical Subject Headings Thesaurus (MeSH) and Boolean operators, using the following keywords: (“Long non‐coding RNAs” “Long non coding RNA” “Long Non‐Protein‐Coding RNA” OR “Long non protein coding RNA” OR “Long ncRNA” OR “Long Non‐Translated RNA” OR “lncRNAs” OR “Lnc RNA” OR “lncRNA” OR “LINC” OR “Long Untranslated RNA” OR “Long ncRNAs” OR “Long Intergenic Non‐Protein Coding RNA” OR “Long Intergenic Non Protein Coding RNA” OR “LincRNAs” OR “LINC RNA” OR “LincRNA”) AND (“biomarker” OR “diagnostic” OR “expression”) AND (“acute myeloid leukemia” OR “AML” OR “Acute promyelocytic leukemia” OR “APL” OR “De novo‐acute myeloid leukemia” OR “de novo acute myeloid leukemia” OR “de novo AML”). A detailed searching strategy is incorporated in File S2.

### 2.2 Inclusion and exclusion strategy

In order to assess the diagnostic accuracy of lncRNAs in AML, we included original human studies conducted on samples obtained from AML patients and healthy individuals. Diagnostic accuracy tests should compare lncRNAs to an established reference standard to determine sensitivity and specificity. Eligible studies only written in English language were included. In addition, studies that meet the following inclusion criteria were included in this study: (1) Studies providing sufficient data to determine diagnostic accuracy tests, such as false positives, false negatives, true positives, and true negatives; (2) Case–control or cohort studies investigating differential lncRNA expression in AML patients; (3) Studies that reported sensitivity, specificity, or area under the curve (AUC) values of lncRNAs for diagnosing AML; (4) Studies with enough original data for statistical analysis of diagnostic information.

The exclusion criteria were presented as follows: (1) Sudies published in language other than English; (2) Duplicate articles; (3) Reviews, expert's opinions, case reports, case series, and meta‐analyses; (4) Studies that did not report all diagnostic parameters; (5) nonhuman studies (animal model studies) were deemed ineligible and, therefore, excluded.

### 2.3 Data extraction

The studies were imported into EndNote 20 software to identify and remove duplicates. The titles, abstracts, and full‐length texts of the selected articles were carefully screened by two independent reviewers based on the eligibility criteria. Two independent authors (YS and EA) extracted data using a standard data collection form. Disagreements between the two reviewers was resolved through discussion, with the involvement of the third reviewer (AB).

The information collected from each articles includes: (1) Basic information such as the first author, year of publication, and country; (2) Study participants: sample size and type of specimen; (3) information on methods: detection method, lncRNA name, expression status of lncRNAs, and reference gene; (4) The outcome: cutoff, area under curve (AUC), and its 95% confidence interval (CI), and diagnostic 4‐grid contingency table: true positive (TP), true negative (TN), false positive (FP), and false negative (FN). The extracted data were cross‐checked by two reviewers (AA and TK). Any disagreements between the data extractors were handled through discussion and consensus through verification.

### 2.4 Risk of bias (quality) assessment

Three authors (AA, TK, and AM) independently assessed the quality of eligible articles. Review Manager 5.4 was utilized to evaluate the quality of the eligible studies using the modified Quality Assessment of Diagnostic Accuracy Studies 2 (QUADAS‐2). The QUADAS‐2 tool is employed for assessing the quality of diagnostic accuracy studies, involving the evaluation of four main domains: patient selection, process and timing, reference standards, and index testing.^27^ The risk was categorized as “low,” “high,” or “unclear”. Any discrepancies were resolved through discussions among researchers. The overall risk of bias for comparison can be assessed by considering the risk of bias for each domain.

### 2.5 Statistical analysis

The collected data were entered into Microsoft Excel software and subsequently exported to Stata version 14.2 (Stata Corporation, College Station, TX, USA) software for statistical analysis. Subsequently, the data were converted into diagnostic numbers, representing TP, FP, FN, and TN. We calculated the overall accuracy of diagnostic tests, including pooled sensitivity, pooled specificity, PLR, NLR, DOR, and area under the curve (AUC), along with corresponding 95% confidence intervals (CI). These calculations were performed to determine the diagnostic value of lncRNAs.

Heterogeneity tests, assessing variability between studies, were conducted by Cochran's Q statistic and *I* ^2^ tests. A value of *I* ^2^ test statistic greater than 50% and a *p*‐value less than 0.05 indicated significant heterogeneity between studies.^28^ The random‐effect model was chosen due to significant heterogeneity between included studies. A bivariate random‐effects model was fitted to estimate the summary receiver operating characteristics (SROC) curve.

Meta‐regression and subgroup analysis were conducted to assess sources of heterogeneity. Subgroup analysis was performed based on sample size, regulation mode, specimen source, reference range, and cutoff value. The results were presented using a forest plot. A sensitivity analysis was performed to determine the stability of the results. Deeks' funnel plot was utilized to assess publication bias. Furthermore, Fagan's nomogram was developed to further evaluate the diagnostic efficacy of lncRNAs. In additions, A *p*‐value less than 0.05 was considered statistically significant.

## 3 RESULTS

### 3.1 Literature search and study characteristics

**FIGURE 1:**
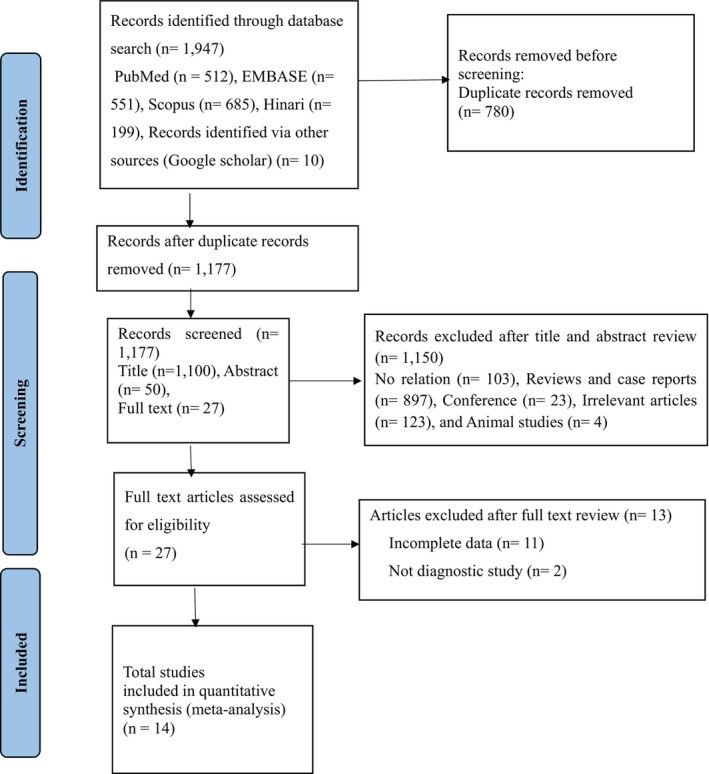
Flow of chart showing selection of studies for meta‐analysis.

We searched a total of 1957 studies in the PubMed, EMBASE, Scopus, and Hinari databases. After excluding 780 duplicate records using EndNote 20 software, we further screened out 1150 studies deemed irrelevant, including conference proceedings, case reports, reviews, animal studies, and those lacking complete data. Following the screening of titles, abstracts, and full texts, we removed 13 studies with incomplete data. Ultimately, our meta‐analysis included 14 eligible articles covering 19 different lncRNAs. The study screening process is presented in a flowchart adhering to PRISMA guidelines (Figure 1). The present study included a total of 1588 AML patients and 529 healthy controls. All studies utilized the quantitative reverse transcription polymerase chain reaction (qRT‐PCR) method to detect the expression of lncRNAs. The majority of the included studies were conducted in China,^20^, ^22^, ^29^, ^30^, ^31^, ^32^, ^33^, ^34^, ^35^, ^36^ four studies conducted in Iran,^37^, ^38^, ^39^ and remaining study was from Africa (Egypt).^40^ Moreover, all included studies were published after 2018 (Table 1).

**TABLE 1:** Characteristics of included studies in meta‐analysis.

Authors	Year	Country	lncRNAs	Expression	Specimen	Method	Reference	AML	HC	Cut off	Sen (%)	Spe (%)	AUC
MA L et al^26^	2018	China	LINC00265	Up	Serum	qRT‐PCR	GAPDH	135	35	4.63	72.43	91.45	0.8131
Sheng H et al^18^	2021	China	LncRNA FBXL19‐AS1	UP	Serum	qRT‐PCR	GAPDH	137	43	NA	81.4	87.8	0.841
Tan Z et al^27^	2021	China	LncRNA ANRIL	Up	BMMC	qRT‐PCR	GAPDH	178	30	NA	84.8	83.3	0.886
Xio Q et al^20^	2022	China	Linc 00265	Down	Plasma	qRT‐PCR	GAPDH	65	20	0.009	85	64.6	0.74
Xio Q et al^20^	2022	China	Linc 00467	Down	Plasma	qRT‐PCR	GAPDH	65	20	0.0104	100	50.8	0.7246
Xio Q et al^20^	2022	China	UAC1	Down	Plasma	qRT‐PCR	GAPDH	65	20	0.0122	90	50.8	0.6623
Xio Q et al^20^	2022	China	SNHG1	Up	Plasma	qRT‐PCR	GAPDH	65	20	0.0227	95	49.2	0.6631
Wang Y et al^28^	2018	China	Linc00899	Up	Serum	qRT‐PCR	GAPDH	153	54	4.67	92.7	83.4	0.807
Abdelrahman AM et al^37^	2023	Egypt	LncRNATUG1	Up	BMMC	qRT‐PCR	β‐Actin	80	20	2.72	88.6	100	0.97
Abdelrahman AM et al^37^	2023	Egypt	lncRNAZEB2‐AS1	Up	BMMC	qRT‐PCR	β‐Actin	80	20	0.26	60.9	100	0.82
Zhang TG et al^29^	2018	China	LncRNA H19	UP	BMMC	qRT‐PCR	NA	161	36	0.121	49.1	80.6	0.655
Yang L et al^30^	2018	China	LncRNA PANDAR	Up	BMMC	qRT‐PCR	NA	119	26	0.84	65.5	80.8	0.800
Jiang Z et al^31^	2019	China	LncRNA MVIH	Up	BMMC	qRT‐PCR	GAPDH	212	70	1.002	86.3	51.4	0.742
He C et al^32^	2020	China	LncRNA MEG3	Down	BMMC	qRT‐PCR	GAPDH	122	30	0.519	83.3	78.7	0.848
Eslami MM et al^34^	2023	Iran	LncRNA NORAD	Up	BMMC	qRT‐PCR	GAPDH	60	49	NA	65	63	0.64
Ganji A et al^35^	2023	Iran	LncRNA AB073614	Up	BMMC	qRT‐PCR	GAPDH	30	20	1.045	96.7	100	0.98
Ganji A et al^35^	2023	Iran	LncRNA FER1L4	Down	BMMC	qRT‐PCR	GAPDH	30	20	0.77	100	100	1
Pashaiefer H et al^36^	2018	Iran	LncRNA IRAIN	Down	BMMC	qRT‐PCR	β‐glucuronidase	64	51	0.0113	63	70.3	0.707
Ruijuan W et al^33^	2023	China	lncRNA RBM5‐AS1	Up	BMMC	qRT‐PCR	GAPDH	72	45	NA	87.5	84.4	0.853

Abbreviations: AML, acute myeloid leukemia; AUC, area under curve; BMMC, bone marrow mono nuclear cells; GAPDH, Glyceraldehyde‐3‐phosphate dehydrogenase; HC, healthy control; NA, not available; qRT‐PCR, quantitative reverse transcription polymerase chain reaction; Sen, sensitivity; Spe, specificity.

### 3.2 Quality assessment

Three authors namely, AA, TK, and AM, independently assessed the quality of the 14 included articles using the QUADAS‐2 quality assessment tool. This tool evaluates four domains: patient selection, index testing, reference standards, and flow and timing. Each domain is assigned a risk bias score of high, unclear, or low. Any discrepancies in the quality assessment were resolved by the third assessor (AB). The results were analyzed using RevMan version 5.4 software.

**FIGURE 2:**
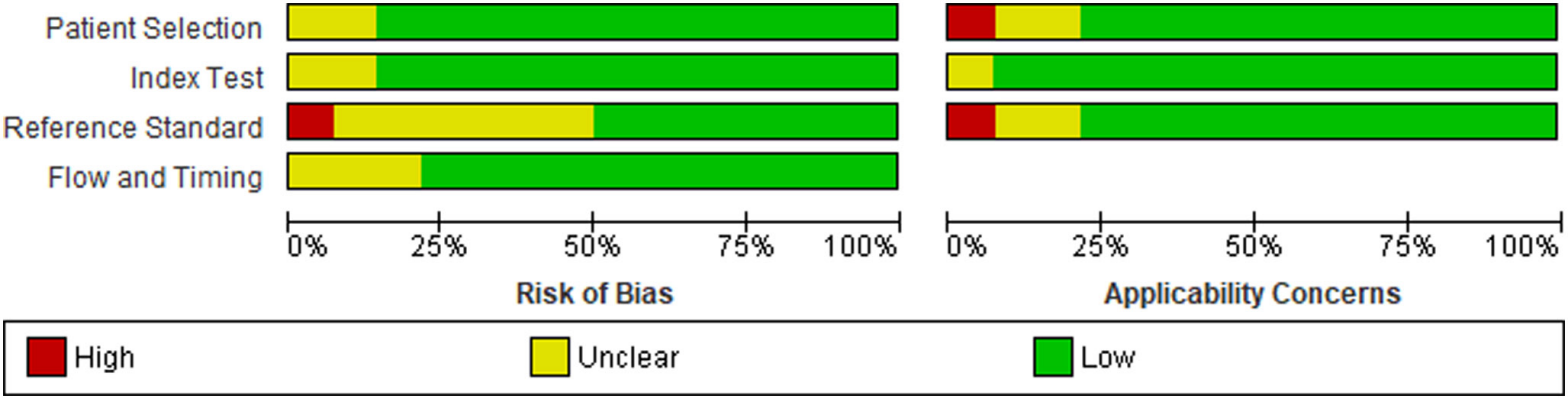
Quality assessment of included studies for diagnostic meta‐analysis using Diagnostic Accuracy studies (QUADAS‐2) tool.

As depicted in Figure 2 below, the majority of studies had a lower risk of bias in the index test domain (86%). In the reference standards and flow and time domains, 50% and 79% of studies had a low‐risk score, respectively. Additionally, most studies showed a low‐risk score in the patient selection and reference standard domains regarding applicability concerns.

### 3.3 Diagnostic accuracy of lncRNAs in AML

**FIGURE 3:**
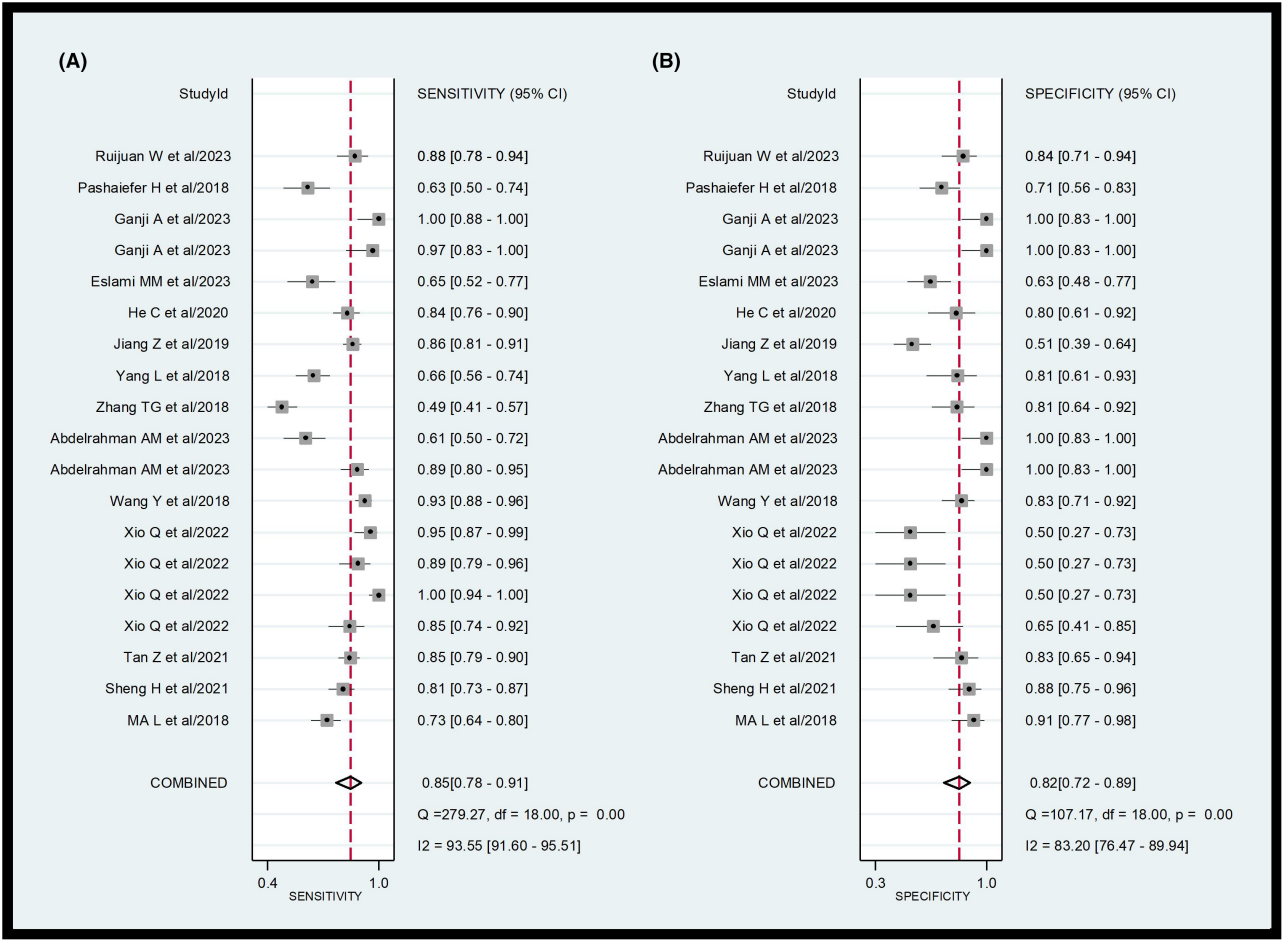
Forest plots of the diagnostic value for the lncRNAs in detecting AML. (A) sensitivity, (B) specificity.

A total of 14 eligible studies were included in the meta‐analysis. The forest plot of the pooled sensitivity and specificity of lncRNAs in diagnosing AML is shown in Figure 3. The pooled diagnostic values are listed as follows: sensitivity 0.85 (95% CI = 0.78–0.91), specificity 0.82 (95% CI = 0.72–0.89), positive likelihood ratio (PLR) 4.7 (95% CI = 2.9–7.4), negative likelihood ratio (NLR) 0.18 (95% CI = 0.12–0.28), and diagnostic odds ratio (DOR) 26 (95% CI = 12–53). As illustrated in the forest plot, significant heterogeneity was observed in the pooled sensitivity (*I* ^2^ = 93.55; 95% CI = 91.6–95.51) and specificity (*I* ^2^ = 83.2; 95% CI = 76.47–89.94). The Q test *p* value was <0.05, suggesting significant heterogeneity among eligible studies. Therefore, a random effects model was employed to estimate the diagnostic performance of lncRNAs in diagnosing AML.

**FIGURE 4:**
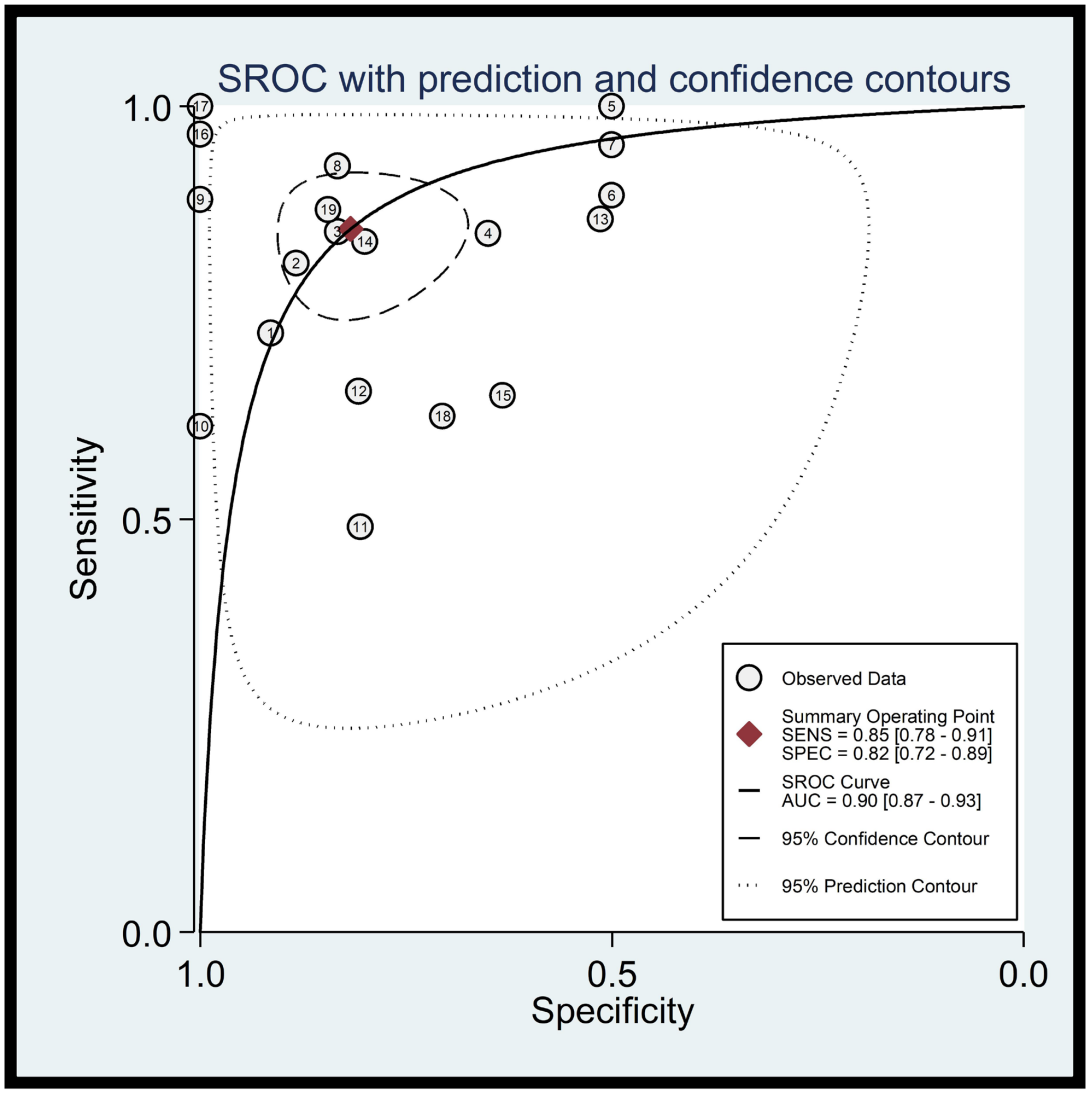
SROC curve of the lncRNAs in detecting AML.

As shown in Figure 4, the Summary Receiver Operating Characteristics (SROC) curve was plotted to assess diagnostic accuracy. The AUC was 0.90 (95% CI = 0.87–0.93), indicating the superior diagnostic value of lncRNAs in the diagnosis of AML.

### 3.4 Subgroup analysis and meta regression

**FIGURE 5:**
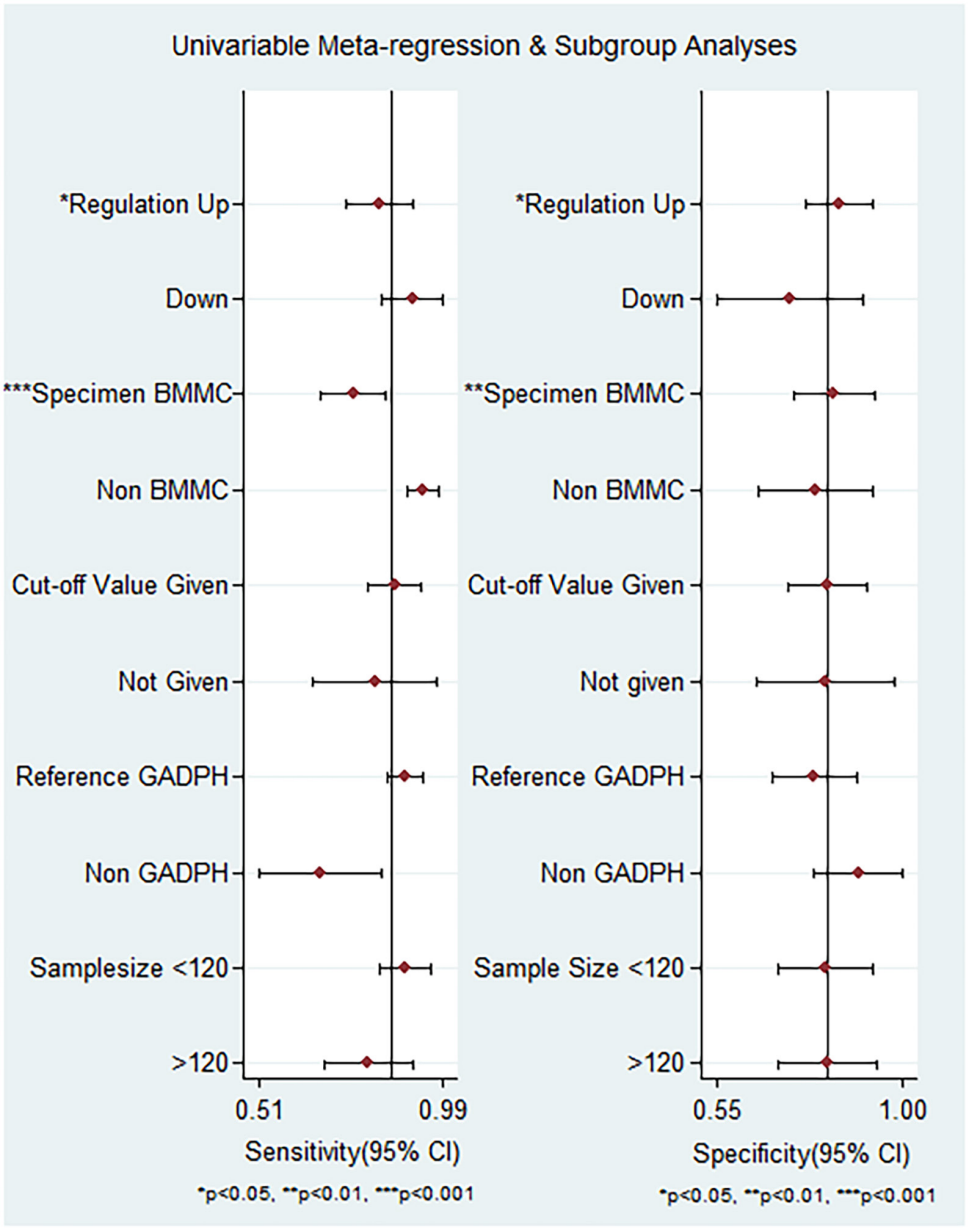
Meta regression analysis * indicates *p* < 0.05, ** indicates *p* < 0.01, and *** denotes *p* < 0.001.

Subgroup analysis and meta‐regression were performed to investigate potential sources of heterogeneity. Subgroups were categorized based on regulation mode, specimen type, cutoff values, reference gene, and sample size. According to the subgroup analysis, the regulation mode and specimen type subgroups found to be associated with heterogeneity (*p* < 0.05). Conversely, the differences in heterogeneity observed in the cutoff values subgroup, reference gene subgroup, and sample size subgroup were not statistically significant (*p* > 0.05) (see Figure 5).

The results of subgroup analysis are provided in Table 2. This study indicates that there is no significant difference observed in the overall diagnostic accuracy of lncRNA between up‐expression and down‐expression statuses of lncRNAs. The diagnostic accuracy of AML for up‐expression and down‐expression lncRNA listed as follows: sensitivity was 0.82 (95% CI = 0.73–0.88) versus 0.93 (95%CI = 0.73–0.98), specificity was 0.85 (95% CI = 0.74–0.92) versus 0.73 (95% CI = 0.53–0.86), PLR was 5.5 (95% CI = 3.0–9.8) versus 3.4 (95% CI = 1.8–6.5), NLR was 0.21 (95% CI = 0.14–0.32) versus 0.1 (95% CI = 0.02–0.46), DOR 26 (95% CI = 11–59) versus 35 (95% CI = 15–245), and AUC was 0.90 (95% CI = 0.87–0.92) versus 0.89 (95% CI = 0.85–0.91), respectively.

**TABLE 2:** Subgroup analysis results of included studies.

Subgroup	No. studies	Sen (95% CI)	Spe (95% CI)	PLR (95% CI)	NLR (95% CI)	DOR (95% CI)	AUC (95% CI)
Regulation mode							
Up	13	0.82 (0.73–0.88)	0.85 (0.74–0.92)	5.5 (3.0–9.8)	0.21 (0.14–0.32)	26 (11–59)	0.90 (0.87–0.92)
Down	6	0.93 (0.73–0.98)	0.73 (0.53–0.86)	3.4 (1.8–6.5)	0.1 (0.02–0.46)	35 (15–245)	0.89 (0.85–0.91)
Specimen type							
BMMC	10	0.76 (0.66–0.83)	0.81 (0.70–0.89)	4.0 (2.4–6.8)	0.3 (0.21–0.43)	13 (6–30)	0.85 (0.82–0.88)
Non‐BMMC	9	0.93 (0.85–0.97)	0.82 (0.63–0.93)	5.2 (2.3–11.7)	0.09 (0.04–0.20)	59 (16–216)	0.95 (0.93–0.97)
Cut‐off values							
Given	15	0.87 (0.77–0.93)	0.83 (0.60–0.91)	5.1 (2.7–9.7)	0.16 (0.09–0.29)	32 (12–86)	0.92 (0.89–0.94)
Not given	4	0.81 (0.72–0.87)	0.81 (0.69–0.89)	4.2 (2.4–7.6)	0.24 (0.15–0.39)	18 (6–49)	0.88 (0.84–0.90)
Reference gene							
GADPH	14	0.89 (0.83–0.93)	0.78 (0.67–0.87)	4.1 (2.6–6.7)	0.14 (0.09–0.23)	30 (13–67)	0.92 (0.89–0.94)
Non GADPH	5	0.67 (0.52–0.79)	0.91 (0.65–0.98)	7.4 (1.4–38.3)	0.37 (0.22–0.61)	20 (2–162)	0.83 (0.79–0.86)
Sample size							
Greater than 120	8	0.79 (0.69–0.87)	0.81 (0.72–0.88)	4.2 (2.8–6.3)	0.26 (0.17–0.38)	16 (8–32)	0.87 (0.84–0.90)
Less than 120	11	0.90 (0.79–0.96)	0.86 (0.63–0.96)	6.5 (2.1–19.8)	0.12 (0.05–0.27)	55 (11–269)	0.90 (0.92–0.96)

Abbreviations: AUC, area under curve; BMMC, bone marrow mono nuclear cells; DOR, diagnostic odds ratio; GAPDH, Glyceraldehyde‐3‐phosphate dehydrogenase; NLR, negative likelihood ration; PLR, positive likelihood ratio; Sen, sensitivity; Spe, specificity.

LncRNAs derived from non‐bone marrow mononuclear cells (BMMC) exhibited higher diagnostic accuracy for AML patients compared to those derived from BMMC. The sensitivity, specificity, PLR, NLR, DOR, and AUC for non‐BMMC lncRNAs were 0.93 (95% CI = 0.85–0.97), 0.82 (95% CI = 0.63–0.93), 5.2 (95% CI = 2.3–11.7), 0.09 (95% CI = 0.04–0.20), 59 (95% CI = 16–216), and 0.95 (95% CI = 0.93–0.97), respectively. While BMMC‐derived lncRNAs had lower diagnostic performance with sensitivity 0.76 (95% CI = 0.66–0.83), specificity 0.81 (95% CI = 0.70–0.89), PLR 4.0 (95% CI = 2.4–6.8), NLR 0.3 (95% CI = 0.21–0.43), DOR 14 (95% CI = 7–30), and AUC 0.85 (95% CI = 0.82–0.88).

Studies with cutoff values demonstrated better diagnostic value when compared to studies without cut‐off values. The pooled values of sensitivity, specificity, PLR, NLR, DOR, and AUC are 0.87 (95% CI = 0.77–0.93), 0.83 (95% CI = 0.60–0.91), 5.1 (95% CI = 2.7–2.7‐9.7), 0.16 (95% CI = 0.09–0.29), 32 (95% CI = 12–86), and 0.92 (95% CI = 0.89–0.94), respectively for studies with cutoff values. In contrast, studies without cutoff values had inferior diagnostic accuracy with pooled sensitivity, specificity, PLR, NLR, DOR, and AUC of 0.81 (95% CI = 0.72–0.87), 0.81 (95% CI = 0.69–0.89), 4.2 (95% CI = 2.4–7.6), 0.24 (95% CI = 0.15–0.39), 18 (95% CI = 6–49), and 0.88 (95% CI = 0.84–0.90), respectively.

Interestingly, most studies selected GADPH as an internal reference. The diagnostic value of the groups using GADPH as the internal reference groups showed better diagnostic accuracy compared to the non‐GADPH group. In the GADPH reference group, the pooled sensitivity, specificity, PLR, NLR, DOR, and AUC were 0.89 (95% CI = 0.83–0.93), 0.78 (95% CI = 0.67–0.87), 4.1 (95% CI = 2.6–6.7), 0.14 (95% CI = 0.09–0.23), 30 (95% CI =13–67), and 0.92 (95% CI = 0.89–0.94), respectively. On the contrary, the non‐GADPH group had lower diagnostic values with sensitivity 0.67 (95% CI = 0.52–0.79), specificity 0.91 (95% CI = 0.65–0.98), PLR 7.4 (95% CI = 1.4–38.3), NLR 0.37 (95% CI = 0.22–0.61), DOR 20 (95% CI = 2–162), and AUC 0.83 (95% CI = 0.79–0.86).

The group with a sample size ≤120 had better diagnostic value than the group with a sample size >120, including sensitivity 0.90 (95% CI = 0.79–0.96), specificity 0.86 (95% CI = 0.63–0.96), PLR 6.5 (95% CI = 2.1–19.8), NLR 0.12 (95% CI = 0.05–0.27), DOR 55 (95% CI = 11–269), and AUC 0.90 (95% CI = 0.92–0.96). In the group with a sample size >120, the corresponding values for sensitivity, specificity, PLR, NLR, DOR, and AUC were 0.79 (95% CI = 0.69–0.87), 0.81 (95% CI = 0.72–0.88), 4.2 (95% CI = 2.8–6.3), 0.26 (95% CI = 0.17–0.38), 16 (95% CI = 8–32), and 0.87 (95% CI = 0.84–0.90), respectively.

### 3.5 Clinical utility of lncRNAs for AML diagnosis

**FIGURE 6:**
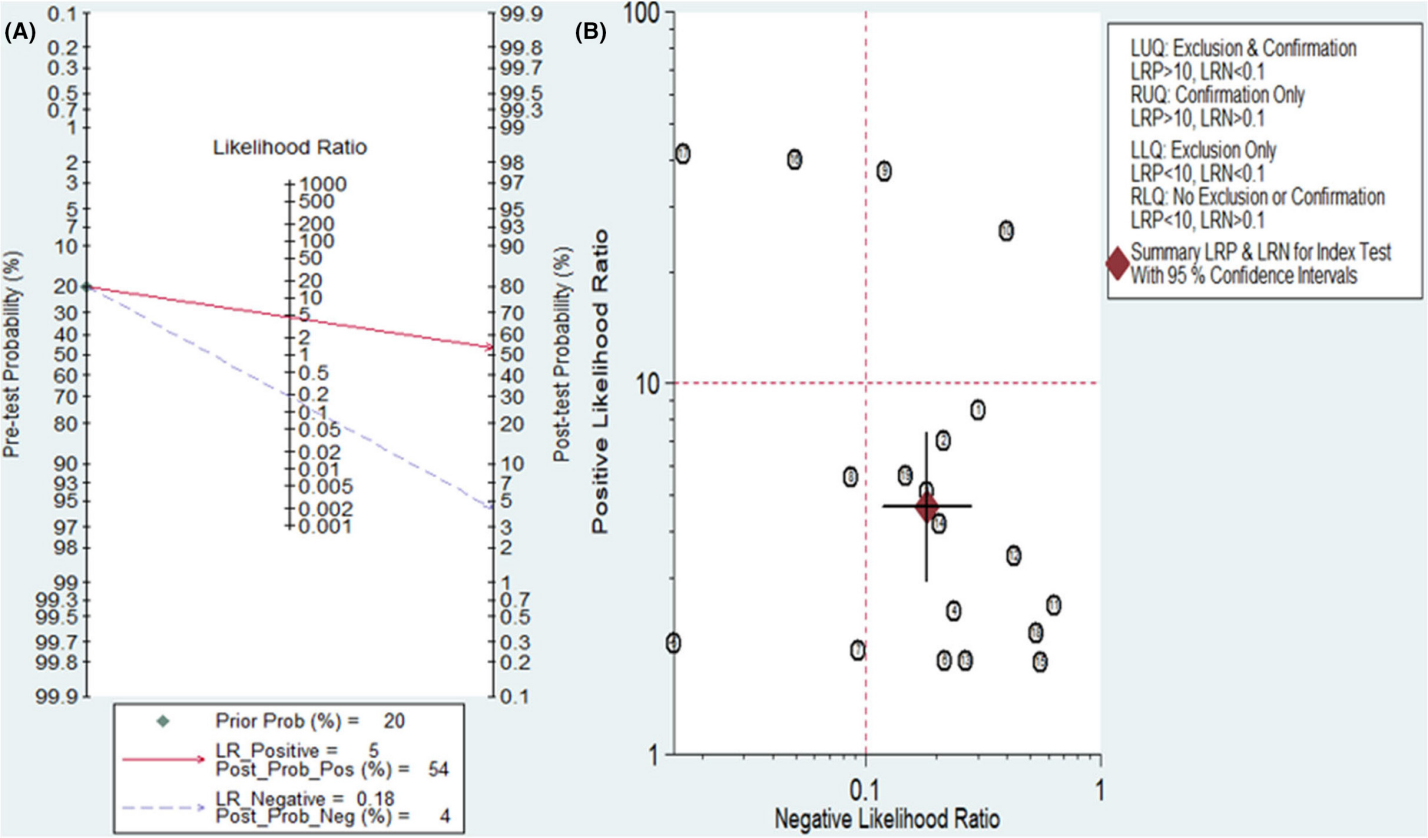
Clinical utility of lncRNAs in the diagnosis of AML. (A) Fagan's nomogram plot of lncRNAs for estimating post‐test possibility. (B) Scattering plot of positive likelihood ratio and negative likelihood ratio when using lncRNA to diagnose AML. LLQ, left lower quadrant; LRN, likelihood ratio negative; LRP, likelihood ratio positive; LUQ, left upper quadrant; RLQ, right lower quadrant; RUQ, right upper quadrant.

The diagnostic value of lncRNAs for AML was illustrated using Fagan's nomogram. As shown in Figure 6A, if a patient had a positive lncRNA result, the posttest probability of individuals suffering from AML would be approximately 54% (indicated by the red line). Conversely, if the test result was negative, the posttest probability that the participant was affected by AML would be approximately 4% (indicated by the blue line). This suggests that lncRNAs are a promising indicator for diagnosing AML.

A likelihood matrix graph was plotted based on the combination of PLR and NLR to determine clinical applicability (Figure 6B). Studies with PLR >10 and NLR <0.1 indicate superior diagnostic accuracy. According to a study by Ganji A. et al., lncRNAs, particularly LncRNA AB073614 and LncRNA FER1L4, have shown the best diagnostic accuracy. As a result, these lncRNAs may be promising for AML diagnosis and should be further investigated in future studies.

### 3.6 Publication bias and sensitivity analysis

**FIGURE 7:**
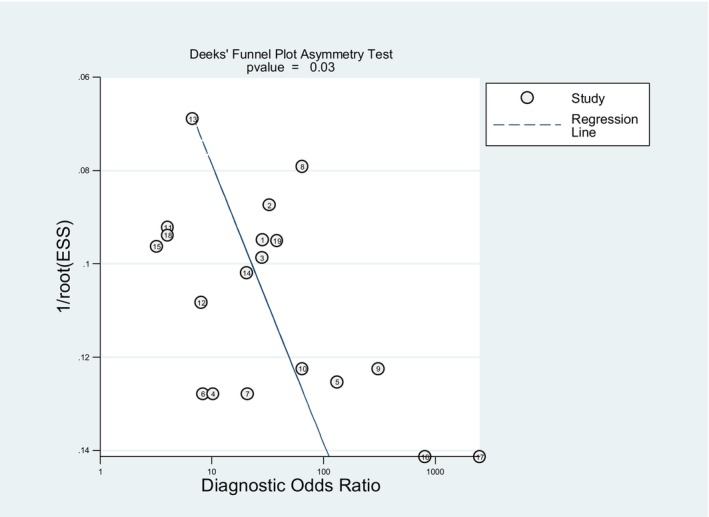
Deek's funnel plot asymmetry test for publication bias.

Deeks' funnel plot asymmetry test was used to determine publication bias in the included studies. The *p* value of Deeks' test was 0.03, indicating the presence of publication bias among eligible studies, as illustrated in Figure 7.

**FIGURE 8:**
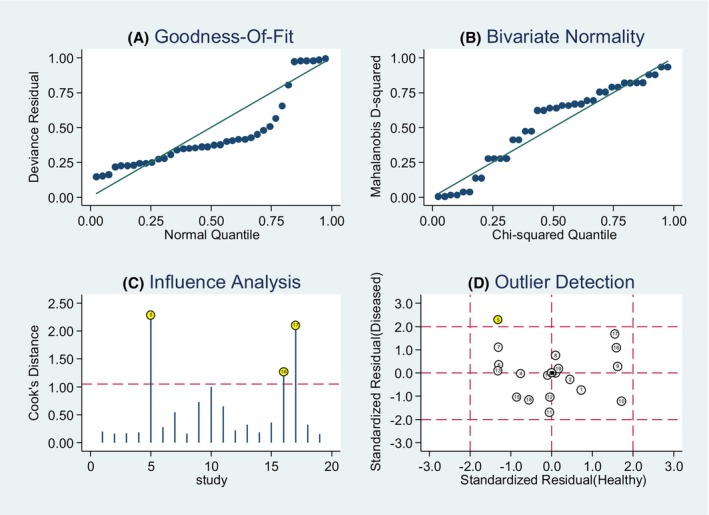
Sensitivity analysis. (A) Goodness of fit, (B) Bivariate normality, (C) Influence analysis, and (D) Outlier detection.

Moreover, we performed a goodness of fit and bivariate normality analysis, demonstrating the robustness of our model was (Figure 8). Sensitivity analysis was performed to detect outliers, with the study by Xio Q. et al. being identified as an outlier. Upon removing the outlier, no significant changes in the pooled diagnostic parameters were observed (Table 3).

**TABLE 3:** Diagnostic performance of lncRNAs in diagnosing AML after outlier is excluded.

Analysis	Overall	Outlier excluded
Number of studies	19	1
Sensitivity (95% CI)	0.85 (0.79–0.91)	0.83 (0.76–0.89)
Specificity (95% CI)	0.82 (0.72–0.89)	0.83 (0.73–0.90)
PLR (95% CI)	4.7 (2.9–7.4)	4.9 (3–8.1)
NLR (95% CI)	0.8 (0.12–0.28)	0.2 (0.14–0.30)
DOR (95% CI)	26 (12–53)	24 (11–53)
AUC (95% CI)	0.90 (0.87–0.93)	0.90 (0.87–0.92)

## 4 DISCUSSION

AML is rapidly fatal and the most common form of acute leukemia, characterized by the clonal expansion of immature myeloid‐derived cells in the BM and blood stream.^41^ The identification of genetic abnormalities in AML focused on protein‐coding genes to provide prognostic value and gain insight into the molecular complexity of AML. Due to the heterogeneous nature of this disease, the underlying molecular mechanisms driving AML development and progression remain unsolved. Dysregulation of lncRNA has been associated with malignant transformation and functions as a potential biomarker for various cancers, including AML.^15^ Unlike other conventional markers, lncRNA offers high sensitivity and specificity, enables early detection, and provides insight into the underlying mechanisms of AML.^25^

This meta‐analysis included a total of 14 articles reporting 19 different lncRNAs with 1588 AML patients and 529 healthy controls. The pooled diagnostic values showed that lncRNAs demonstrate high sensitivity (0.85; 95% CI = 0.78–0.91) and specificity (0.82; 95% CI = 0.72–0.89) for diagnosing AML. The pooled PLR of 4.7 indicated the probability of subjects diagnosed with AML increasing by 4.7‐fold when lncRNA detection was positive. The NLR value was 0.18, implying only an 18% probability of individuals being diagnosed with AML if the lncRNA test was negative. DOR was employed to assess the discriminative effect of lncRNAs in the diagnosing AML. DOR is an index for discriminating test performance^42^ and DOR >1 indicates a better diagnostic test. In this study, the DOR value was 26, which indicated lncRNA can effectively discriminate between AML patients and healthy individuals. The overall diagnostic accuracy was summarized by the SROC curve, with an AUC of 0.90, representing superior diagnostic performance of lncRNAs for AML. Considering all diagnostic parameters together, these findings strongly indicate that circulating lncRNA has the potential to serve as a diagnostic marker for AML. Similarly, different meta‐analyses have indicated the potential of lncRNA as a diagnostic marker for various conditions, including head and neck squamous cell carcinoma,^43^ melanoma,^44^ hepatocellular carcinoma,^45^ stomach cancer,^46^ and multiple myeloma.^47^ This phenomenon exhibited the capacity of lncRNA to play a role in the regulation of gene expression and malignant transformation.^8^, ^10^

It is noteworthy that there was observed heterogeneity among included studies. Hence, the influence of this confounding factors was examined through meta regression and subgroup analysis. Subgroups of sample size less than 120 demonstrated better diagnostic accuracy than sample size greater than 120, with an AUC of 0.90 (95% CI = 0.92–0.96) versus 0.87 (95% CI = 0.84–0.90), respectively. Similarly, a study by Cao F et al.,^46^ reported superior diagnostic accuracy for sample size less than or equal to 100. This difference might be attributed to the fact that most of the included studies had a sample size of less than 120. On the contrary, the regulation status of lncRNAs did not show a significant diagnostic difference, with an AUC of 0.90 (95% CI = 0.87–0.92) for upregulation status and 0.89 (95% CI = 0.85–0.91) for downregulation status. Another study by Cao F et al., reported superior diagnostic accuracy of upregulation mode of lncRNA.^46^

The detection of blood sample lncRNAs indicated higher diagnostic accuracy than lncRNAs extracted from BMMC, with an AUC value of 0.95 versus 0.85. Consistently, different studies reported higher diagnostic accuracy for blood‐based samples (serum and plasma).^43^, ^48^ Circulating lncRNAs can be identified easily in serum, plasma, whole blood, urine, and other body fluids.^23^, ^24^ On the contrary, different studies showed higher diagnostic performance of lncRNAs from serum samples than plasma.^46^, ^49^ This might be attributed to the release of lncRNAs from blood cells (such as platelets) during coagulation.^49^ However, the optimal specimen for detecting lncRNA expression among AML patients has not been well explored.

On the contrary, the subgroup analysis by cutoff values showed that studies with an optimal cutoff value represented remarkable diagnostic efficacy compared to studies not reporting a cutoff value (AUC of 0.92 versus 0.88). This might be due to the varying number of studies and lack of standardized cutoff values, which may introduce heterogeneity and potentially affect overall diagnostic accuracy.

All included studies employed qRT‐PCR method for lncRNA detection, in which the RNA strand is reverse transcribed into complementary DNA (cDNA). cDNA serves as a template for DNA amplification. This method is emerged as optimal choice for detecting lncRNAs with high sensitivity and specificity.^50^, ^51^, ^52^ In the qRT‐PCR method, an appropriate internal reference gene should be chosen for normalization.^49^ Hence, GADPH is the most commonly used internal reference gene in the included studies. With an AUC of 0.92, lncRNAs demonstrated better diagnostic accuracy when GADPH internal reference for quantitative analysis. On the contrary, Lee C et al.,^53^ identified the top five reference genes (ACTB, UBE2D2, B2M, and RPL37A) for qRT‐PCR normalization in AML using Genorm and Normfinder software. Based on findings of this study SRP14 + B2M was suggested as the best reference gene for normalization in studies involving AML and HCs. However, the selection of reference gene may depend on the sample type. In contrast, different studies have reported GADPH as a least suitable reference gene in different cancer types.^45^, ^54^ Lack of a universally accepted housekeeping control gene for lncRNA, along with ongoing controversy over selecting a suitable reference gene, complicates standardization.

For clinical applicability, Fagan's plot was employed to assess lncRNAs as a diagnostic marker for estimating the probability of individuals being diagnosed with AML. The outcomes of Fagan's nomogram indicated a promising result with post‐test probabilities, showing PLR and NLR values of 0.54 and 0.4, respectively, when the pretest possibility was set at 20%. According to this finding, patients had a 54% probability of developing AML when samples tested positive for the presence of lncRNA. Conversely, when samples tested negative for lncRNA, the post‐test probability of developing the disease reduced to 5%. These findings highlight the potential of lncRNA testing as a valuable and precise tool for diagnosing AML. Therefore, future research should aim to further validate and expand upon these findings in this specific context.

This meta‐analysis has several strengths. It is the first to conduct a detailed evaluation of the diagnostic potential of lncRNAs for effectively diagnosing AML. This finding offers new prospect for developing biomarkers for AML diagnosis. This meta‐analysis conducted an in‐depth assessment of lncRNAs, including meta‐regression and subgroup analysis, to explore factors such as sample size, sample type, regulation mode, and reference gene. This comprehensive approach aimed to assess and elucidate the sources of heterogeneity in the findings.

Our study has certain limitations that should be highlighted. Firstly, ethnic bias may occur because most of the included studies were done in Asian populations (China and Iran). Secondly, the included studies used different cutoff values for lncRNAs, potentially contributing to heterogeneity. Due to the limited number of studies performed outside China, we were unable to conduct subgroup analysis based on ethnicity. The articles written in English were included in this meta‐analysis, which may introduce unavoidable bias. On the contrary, there is no consensus on the selection of stable and uniform internal reference genes, leading to inconsistent results in the quantitative analysis of lncRNAs. Furthermore, most studies used a small sample size, which has limited statistical power. A specific single lncRNA or lncRNA panel as the best diagnostic marker for AML was not identified due to the absence of a large number of similar lncRNAs to pool the results. Because of the limitations mentioned above, these results should be interpreted carefully.

In conclusion, this meta‐analysis suggests that lncRNAs have a significant value in predicting AML. Non‐BMMC‐derived lncRNAs had superior diagnostic potency when compared to BMMC lncRNAs. Furthermore, using a cutoff value and GADPH as a reference gene showed higher diagnostic value compared to their counterparts. Consequently, lncRNAs might be utilized as noninvasive biomarkers for AML patients. However, well designed multicenter and prospective studies with large sample size should be conducted to confirm our results in the future.

## AUTHOR CONTRIBUTIONS

**Yenealem Solomon:** Conceptualization (lead); data curation (lead); formal analysis (lead); funding acquisition (lead); methodology (lead); project administration (lead); validation (lead); writing – original draft (lead); writing – review and editing (lead). **Ayenew Berhan:** Conceptualization (equal); data curation (equal); funding acquisition (equal); methodology (equal); software (equal); supervision (equal); visualization (equal); writing – original draft (equal); writing – review and editing (equal). **Andargachew Almaw:** Data curation (equal); formal analysis (equal); investigation (equal); project administration (equal); validation (equal); writing – original draft (equal). **Tamirat Ersino:** Data curation (equal); formal analysis (equal); project administration (equal); supervision (equal); validation (equal); writing – review and editing (equal). **Shewaneh Damtie:** Funding acquisition (equal); investigation (equal); methodology (equal); software (equal); validation (equal); writing – review and editing (equal). **Teklehaimanot Kiros:** Formal analysis (equal); investigation (equal); resources (equal); software (equal); supervision (equal); visualization (equal); writing – original draft (equal). **Alemie Fentie:** Data curation (equal); formal analysis (equal); methodology (equal); resources (equal); software (equal); validation (equal). **Ermias Sisay Chanie:** Data curation (equal); formal analysis (equal); methodology (equal); project administration (equal); software (equal); supervision (equal); writing – review and editing (equal). **Anteneh Mengist Dessie:** Data curation (equal); formal analysis (equal); investigation (equal); methodology (equal); resources (equal); visualization (equal); writing – review and editing (equal). **Ermiyas Alemayehu:** Conceptualization (equal); data curation (equal); formal analysis (equal); investigation (equal); methodology (equal); project administration (equal); software (equal); supervision (equal); validation (equal); visualization (equal); writing – original draft (equal); writing – review and editing (equal).

## FUNDING INFORMATION

The author's do not have received any specified fund for this project (no funding).

## CONFLICT OF INTEREST STATEMENT

Author(s) declared no potential conflicts of interest with respect to the research, authorship, and/or publication of this article.

## Supporting information


Appendix S1.

## Data Availability

The original data in which conclusion was drawn is available at corresponding author upon reasonable request.
